# Analysis of Reasons for Orthopaedic Walkouts from the Emergency Department in a Private Tertiary Care Centre

**DOI:** 10.5704/MOJ.2011.021

**Published:** 2020-11

**Authors:** KD Roy, GM Sharma, F Qureshi, F Wadia

**Affiliations:** 1 Department of Trauma and Orthopaedics, Royal Gwent Hospital, Newport, United Kingdom; 2 Department of Orthopaedics, MGM Medical College Kamothe, Kamothe, India; 3 Department of Trauma and Orthopaedics, Barnsley Hospital NHS Foundation Trust, Barnsley, United Kingdom; 4 Department of Orthopaedics, Sir HN Reliance Foundation Hospital and Research Centre, Mumbai, India

**Keywords:** orthopaedic, discharge, DAMA, developing countries, cost

## Abstract

**Introduction:**

A small proportion of patients presenting to the Emergency department (ED) of any hospital tend to take discharge against medical advice (DAMA) due to several patient related or hospital/service related reasons. Amongst these, orthopaedic patients are a special group due to their inability to mobilise independently due to injuries and have treatment needs which involve higher costs. The aim of the current study was to ascertain and analyse the reasons for orthopaedic walkouts at a tertiary care new private hospital.

**Materials and Methods:**

This retrospective telephonic structured interview-based study was carried out on all orthopaedic patients taking DAMA during a one-year period from July 2016 to June 2017. They were telephonically interviewed with a structured questionnaire. Hospital and ED records were analysed for demographic as well as temporal characteristics.

**Results:**

A total of 68 orthopaedic patients walked out of casualty against medical advice out of a total 775 (8.77%) orthopaedic patients presenting during the period as against 6.4% overall rate of DAMA for all specialties. The main reasons for DAMA were financial unaffordability of treatment (36.7%), preference for another orthopaedic surgeon (22%) and on advice of the patient’s General Practitioner (16.1%).

**Conclusion:**

Unaffordability of treatment is a significant cause for walkouts amongst orthopaedic patients. Private hospitals need to recognise and implement processes by which these patients can be treated at affordable costs and with coverage either by medical insurance or robust charity programs. Patient education and awareness are important to encourage them to have insurance coverage.

## Introduction

A significant proportion of patients who present to the emergency department take their own discharge against the advice of nursing and medical staff. Of particular concern are those patients who refuse treatment or hospital admission. Although patients are under no obligation to follow medical advice, it is crucial that they are able to understand the implications of this decision after having been explained all the possible consequences^1^.

The pattern of refusal of treatment in developing countries has been of much interest in recent years^2^. In India, it is observed that medical treatment is primarily dictated by the affordability of treatment in the private health sector. This usually directs a large chunk of patients away from private hospitals to public hospitals which offer more affordable treatment. Additionally, the majority of people do not subscribe to health insurance and amongst those who do, their insurance policies may not be accepted at all hospitals^3^. This may lead patients to seek medical care where individual insurance plans can be processed.

Another very common reason for refusal of treatment is the fact that the patient’s “family physician” may dictate where the patients get treated ultimately. This appears to be a unique scenario in urban healthcare in India and could not be verified with relevant literature.

The aim of the present study was to determine the number of Orthopaedic walkouts and analyse the reasons for refusal of treatment among these, after presenting to the emergency room (ER).

## Materials and Methods

After approval by the Institutional Review Board, a descriptive retrospective study was carried out at our newly established tertiary care corporate hospital in Mumbai, India. This institute has 345 inpatient beds and the emergency department has a dedicated 24 hours staff including Emergency medicine doctors, nurses and technicians with independent radiograph and CT facilities within the ER.

**Fig. 1:**
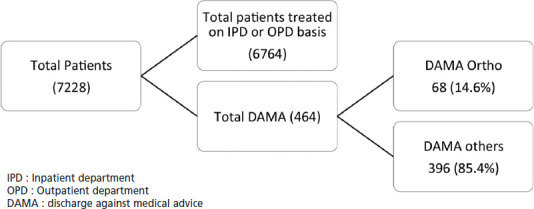
Patient flow to the Emergency Room.

Medical records of all patients visiting the hospital’s emergency department for a period of 12 months from July 2016 to June 2017, were reviewed. A total of 7,228 patients visited the ER during the above-mentioned period, out of which 775 were classified as having a musculoskeletal injury / orthopaedic complaint (e.g. acute back pain or infection). Orthopaedic Patients who took discharge against medical advice were the subject of the current study and their electronic medical records were evaluated in further detail. Fig. 1 depicts the patient flow in the emergency room.

These patients/relatives were contacted telephonically with a Structured interview to ascertain the reasons for walkout, the final place of treatment, the reason for choosing that place and details of final treatment availed / offered. While we understand that an open ended face to face interview, along with transcription of this data and doing a thematic qualitative analysis would have been an ideal research methodology for an in-depth analysis of reasons for DAMA, this was thought not to be practical as it would be difficult to engage with this cohort of patients who had chosen to take treatment elsewhere. Hence it was decided to use a structured telephonic interview technique with some open-ended questions.

An attempt to contact each patient was made for a maximum of 3 occasions at different times. An informed consent was verbally obtained from each participant before commencing the interview. The interview included a pre-decided set of questions, however the patients and relatives were allowed to talk about their treatment freely and the answers were noted as free text either during the call or immediately after the call ended.

Analysis of data was done ensuring strict patient anonymity. Demographic characteristics were analysed to know the age and sex distribution. The time of arrival of the patients was noted to classify the patients as daytime (0800 to 2000 hrs) or night-time (2000 to 0800 hrs).

The data set from records and the interviews were summarised using simple descriptive statistics. Certain comparative data were subjected to statistical analysis using Chi square test (SPSS version 20.0).

## Results

During the study period, a total of 7,228 patients visited the ER at our hospital. 775 patients were found to have musculo-skeletal injuries/orthopaedic complaints. It was observed that a total of 464 patients refused treatment advised in the ER and took discharge against medical advice (DAMA). 68 patients among these were found to have orthopaedic complaints or injuries.

Demographic characteristics of these patients are shown in Table I. Males accounted for a vast majority of patients taking DAMA (72.1%) and 88% were adults with a mean age of 45.36 years (Median = 41±20).

**Table I:** Demographic characteristics of Orthopaedic patients going discharge against medical advise

Character/Variable	Number	Percentage
Sex		
Male	49	72.1%
Female	19	27.9%
Age group		
Paediatric (<16 years)	8	11.7%
Adult (16 and above)	60	88.3%
Limb/region injured		
Upper limb	42	61.8%
Lower limb	23	33.8%
Spine	3	4.4%
Timing of DAMA		
Day (08:00 to 20:00)	33	48.6%
Night (20:00 to 08:00)	35	51.4%

Orthopaedic patients accounted for 10.7% of the total patients coming to the ER. Among those who took DAMA, 14.62% were orthopaedic patients. The monthly distribution of patients coming to the ER is presented in (Table II). The percentage of DAMA amongst orthopaedic patients was higher at 8.77% (68 out of 775) as compared to an overall DAMA rate of 6.4% (464 out of 7,228) and 6.14% (396 out of 6,453) for non-orthopaedic patients. This difference was found to be statistically significant (Chi square test [p value (2 tailed) = 0.004646] and Odd’s ratio [OR=1.471]) and has been illustrated in Table III.

**Table II:** Monthly figures for Orthopaedic and overall walkouts from the Emergency Room

Month	Ortho DAMA	Total DAMA	Total Ortho	Total ER
August 2016	7	46	60	611
September 2016	8	47	67	739
October 2016	11	49	66	827
November 2016	6	39	68	596
December 2016	7	28	55	356
January 2017	3	36	64	572
February 2017	6	33	72	545
March 2017	3	35	50	621
April 2017	2	34	74	552
May 2017	5	35	63	593
June 2017	4	34	77	647
July 2017	6	48	59	569
Total	68	464	775	7,228

**Table III:** Single Table Analysis of Total Patients going discharge against medical advise versus Total Patients not going discharge against medical advise

	DAMA	Non - DAMA	Total
Orthopaedic Patients	68	707	775
Non-Orthopaedic Patients	396	6057	6453
Total	464	6764	7228

Mid-P exact p-value(2-tail) – 0.004646 , Odd’s ratio – 1.471

**Fig. 2:**
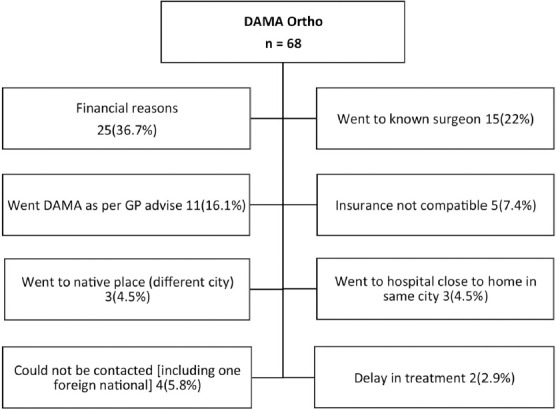
Reasons for discharge against medical advice (DAMA).

**Fig. 3:**
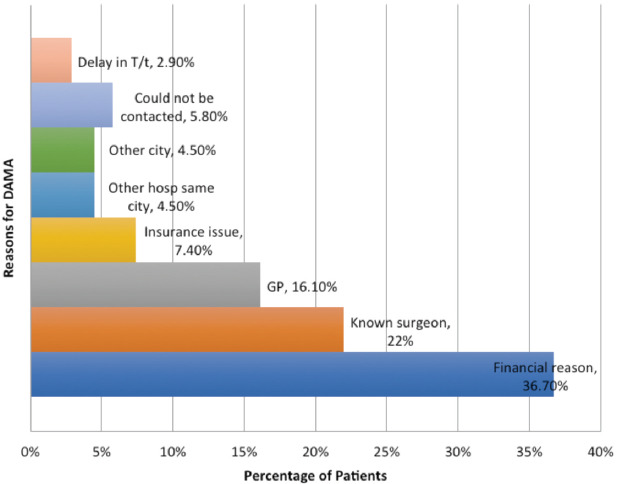
Bar chart depicting percentage distribution of reasons for DAMA.

The most commonly cited reasons for DAMA were financial unaffordability (36.7%), seeking treatment with a known surgeon/family orthopaedic surgeon (22%) and as advised by family physician (16.1%). The various reasons given by patients along with frequency/percentage have been laid out in Fig. 2 and 3.

Fifty-nine of the sixty-eight patients taking DAMA were found to have sought treatment at a different hospital in the same city. Close to 75% of these (n=44) were ultimately treated at another private sector hospital and only 25% (n=15) reported as having been treated at a public sector/government hospital.

## Discussion

There is a great variability of walkout rates in published literature ranging from 0.06% to 20%^4-8^. The overall DAMA rate from the emergency room at our hospital over one year was 6.4%. Hadadi *et al* found 12.8% patients left their ER at a University hospital in Tehran^8^ while only 0.7% went DAMA in a Dutch study by Linden *et al*^7^.

There appears to be a clear-cut divide in the reasons for DAMA in the developed countries versus the developing nations. A literature review by Clarey *et al* in 2012 shows the most common reasons for DAMA to be long waiting time, poor communication during waiting and overcrowding of ED^9^.

The low rate of DAMA observed by Linden *et al* was attributed to milder ED crowding in the Netherlands as compared to other countries^7^. They have however gone on to elaborate that they observed high ‘target time to treatment elapse rate’ and the main reason for patients to walkout was waiting time, suggesting that crowding was an important issue in their study as well. Conversely delay in treatment/waiting time was the least quoted reason for DAMA in our study (n=2, 2.9%).

An Indian study by Naderi *et al* performed at a private hospital in West Bengal, India investigated the role of financial constraints as a reason for patients leaving the ED^10^. Though their overall DAMA rate was 3.8% which is less than many studies in literature the percentage of people citing ‘financial factors’ as a reason for leaving the hospital was a notably high rate of 84%.

Nasir *et al* in a Nigerian study found an overall DAMA rate of 4.2% compared to 6.4% reported in our study^11^. They further found that 43.6% of the patients who took DAMA wished to seek alternate medical care and a further 29.1% mentioned financial constraints as the reason for their self-discharge.

Financial reasons are usually not relevant in countries where the healthcare system is largely either government sponsored or in those countries with a strong insurance-based system, with overcrowding and waiting time being more important issues^12-16^.

A Nigerian study by Ngim and colleagues delved into the reasons for DAMA specifically amongst orthopaedic patients^17^. The most common reason observed by them was ‘leaving for treatment by traditional bone setters’ (37.9%). This brings out a unique facet of medical practises in this part of the world. The authors concluded that an important preventive strategy would be to intensify the campaign to educate society, so that the misplaced belief in supernatural prowess of bone-setters is uprooted.

Interestingly Gunduz *et al* analysed the reasons for DAMA in paediatric emergency services and found restriction on family companions staying with the patients and lack of confidence in therapy as the most common reasons for walkouts^12^.

The percentage of orthopaedic patients leaving the ER in the present study was 8.7% which was higher than the overall walkout rate of 6.4%. ‘Financial reasons’ or unaffordability was the most commonly cited reason for DAMA in the present study and accounted for 36.7% of the walkouts. This is considerably less as compared to the observations by Naderi *et al* (84%) though one can debate that the two cohort samples are not comparable (Orthopaedic DAMA in present study versus DAMA in all patients in the study by Naderi *et al*^10^) and the variability of practises in each region.

Other strong reasons for DAMA noted in the present study include ‘treatment by known surgeon’ and ‘GP/family physician’s advice’ which, respectively accounted for 22% (n=15) and 16.1% (n=11) of the orthopaedic DAMA patients. This highlights a peculiar feature of practise in urban India wherein patient preference for treatment is guided by the advice given by general practitioners and not the hospital per se. When seeking specialist treatment, the patients rely heavily on the advice from their GPs who in turn are affiliated to certain specialists and hospitals.

Menendez *et al* studied the factors associated with self-discharge in orthopaedic in-patients in the United States of America over a 10 years period and found that self-discharges were significantly higher at larger centres located in urban settings^18^. Other patient characteristics associated with higher rates of DAMA were upper limb injury, low household income and no insurance coverage. These findings are similar to our study and show the universality of reasons and characteristics for orthopaedic walkouts.

Orthopaedic patient cohort is a special subset among the patients visiting the ER because a vast majority of them have been involved in various forms of accidents/trauma and are limited in ambulation. Furthermore, treatment in this group often involves the use of implants/prosthesis which adds up to the cost of treatment.

Limitations of this study include it being a single centre study which may have affected the sample size and type of patients, retrospective data collection, recall bias of patients and the lack of a validated questionnaire for gathering information from the patients. Our centre is a private hospital and has been established fairly recently, this fact may affect patients’ decision-making, especially when deciding about surgical treatment.

Though individual predictors for patient walkout are varying and unreliable, steps are needed to ensure that patients do not take DAMA due to financial causes alone. This can be overcome with robust charity programmes or education of the general public about the importance of health insurance.

## Conclusion

Our study highlights that financial unaffordability, patient preference and patient’s general practitioner’s opinion constitute the three main reasons for orthopaedic walkouts in a private healthcare setup.
